# Bilateral Angle Recession and Chronic Post-Traumatic Glaucoma: A Review of the Literature and a Case Report

**DOI:** 10.3390/life13091814

**Published:** 2023-08-27

**Authors:** Valeria Iannucci, Priscilla Manni, Ludovico Alisi, Giulia Mecarelli, Alessandro Lambiase, Alice Bruscolini

**Affiliations:** Department of Sense Organs, Sapienza University of Rome, 00185 Rome, Italy; valeria.iannucci@uniroma1.it (V.I.); priscilla.manni@uniroma1.it (P.M.); ludovico.alisi@uniroma1.it (L.A.); giulia.mecarelli@uniroma1.it (G.M.);

**Keywords:** angle recession, post-traumatic glaucoma, airbag eye injuries, bilateral eye injuries

## Abstract

Ocular trauma affects millions of people worldwide and is a leading cause of secondary glaucoma. Angle recession is the main cause of post-traumatic glaucoma after blunt eye trauma, and it is usually unilateral. The aim of this paper is to investigate the possible causes of angle recession with a bilateral presentation. Airbag activation during traffic accidents is a likely cause to be ruled out, along with repeated head or eye trauma, due to contact sports or a history of physical abuse. These aspects can aid in early detection, appropriate management, and improved outcomes for patients with ocular trauma. Finally, we report the case of a 75-year-old Caucasian man who developed a bilateral angle recession after an airbag impact, with advanced glaucoma in the right eye and ocular hypertension in the left eye. To our knowledge, this is the first case in the literature of chronic post-traumatic glaucoma probably caused by an airbag.

## 1. Epidemiology of Ocular Trauma

Ocular trauma is an important cause of visual impairment and disability, affecting millions worldwide. Approximately 55 million eye injuries occur yearly. Of these, 19 million ocular injuries will result in unilateral vision impairment or blindness and 1.6 million in bilateral blindness [1].

### 1.1. General Risk Factors

The risk of eye injury by age has a bimodal pattern across the lifespan. The first peak occurs in young people (5–25 years, especially in children aged 5–8), the second in the elderly (>70 years). Male gender is strongly associated with eye trauma (male/female ratio up to 8:1) [1]. The difference by gender is striking in young people and tends to disappear in the elderly. Low socioeconomic status and poor workplace conditions are also related to an increased risk of eye injury [1].

Further epidemiological insights into ocular trauma will be provided by an ongoing international multicenter clinical registry: the International Globe and Adnexal Trauma Epidemiology Study (IGATES). IGATES is a recent web-based platform collecting data from 37 centers worldwide, promoted by the Asia Pacific Ophthalmic Trauma Society, which will be useful in directing future research on ocular trauma [2]. Along with chemicals, radiation, and heat exposure, mechanical trauma is the primary cause of eye injury [3,4] (Table 1).

Eye trauma can occur in many settings, such as recreational or occupational activities, during a physical assault, or in a motor vehicle accident. Over the last few decades, one particular category of eye trauma that has received particular attention is airbag-related eye injury.

### 1.2. Airbag-Related Eye Injuries

During the Third Global Ministerial Conference on Road Safety in 2020, a goal was set to halve road fatalities by 2030.

Since the 1970s, road fatalities have fallen dramatically in high-income countries, thanks to a combination of efforts: strengthening road safety regulations, improving road design, educating road users, and designing increasingly safe vehicles. Regrettably, low-income countries, which have not fully leveraged the advantages of technology, continue to experience significantly higher death rates compared to high-income countries.

The technologies contributing most to the reduction in road fatalities are ABS (anti-lock braking system), seat belts, child seats, electronic stability control, and airbags [5,6,7].

Airbags are inflatable balloons stored in the steering wheel and frontal dashboard, containing sodium azide powder. During a road accident, they are meant to act like a cushion between the car occupant and the hard structures inside the vehicle. Airbags can decrease the risk of death in a motor vehicle accident by up to 32% in adults, especially with the use of seat belts [8]. Airbag-associated morbidities and fatalities in children under 10 years overcome the protective effect [9]; therefore, device disconnection is strongly recommended when children travel in the front seat.

When car sensors detect a significant deceleration, within 15 msec from a collision, the sodium azide powder ignites, producing a gas solution that inflates the balloon within 35–50 ms Rapid deflation is granted by vents on the balloon’s surface when fully inflated. Correct timing of inflation and deflation of the airbag is crucial to protect the car user. If the bag deploys too soon, it will be deflated when the occupant impacts the steering wheel. On the other hand, if the balloon deploys too late, it will strike the occupant violently during inflation. In this case, the hard impact can result in airbag-related injuries (ARIs) [10,11].

Available data show that ARIs tend to be more severe in unbelted car occupants and are more likely to occur in drivers who sit closer to the steering wheel. According to the literature, most ARIs in adults are classified as minor, and they probably would have been more serious if the airbag were disconnected. The face is the most common site of ARIs (42%), and the eyes are particularly exposed [10].

Airbag deployment can damage the eye through blunt trauma, penetrating trauma, and chemical burn.

Blunt trauma is due to the direct impact of the airbag on the eye: it can result in an ocular contusion, or even globe rupture, if the force of the inflating airbag overcomes the resistance of the eyewall. The anterior segment is more commonly involved in ocular contusion than the posterior segment [12]. Corneal abrasion, endothelial cell loss, corneal flap dislocation after laser in situ keratomileusis (LASIK), traumatic hyphema, angle recession, cyclodialysis, lens dislocation, and traumatic cataract can be found [11]. Posterior segment involvement includes vitreous hemorrhage, retinal edema, retinal break or detachment, traumatic macular hole, Purtscher retinopathy, chorioretinitis sclopetaria, optic nerve avulsion, and traumatic optic neuropathy [11]. Globe avulsion with transection of the optic nerve and the extraocular muscles has been described [13].

Penetrating trauma usually occurs when the inflating airbag shatters eyeglasses, or when a sharp object interposes between the airbag and the car occupant. On the other hand, airbag deployment seems to protect the eye from windshield fragments [14].

The chemical burn may result from alkaline byproducts released during ignition of sodium azide powder inside the airbag: corneal epithelial defect is the most common finding [11], but limbal ischemia has also been described [12].

Specific experimental models have been proposed to understand airbag-induced ocular trauma features better [15,16,17,18,19,20,21,22,23].

Fukagawa sutured porcine eyes into cotton-filled metal orbits in a crash test dummy. Different types of airbags were set to strike the dummy’s face at different distances. This model demonstrated a correlation between airbag deployment and endothelial cell loss [16]. A similar attempt was made using defrosted cadaver heads, but minimal ocular damage was found [17]. However, these experiments were considered insufficiently reliable, because soft tissues lose their biomechanical properties after death. Therefore, three-dimensional finite element analysis (3D-FEA) models of human eyes have been proposed to better demonstrate the effects of airbag-related trauma. These studies show that deformation mostly involves the anterior segment, with any axial length; scleral and corneal wounds are most common at high impact velocities (50–60 m/s) [18], but globe rupture can occur at lower velocities (20–40 m/s) in eyes that have undergone previous surgery, such as trans-scleral IOL fixation [19,20], radial keratotomy [23], and trabeculectomy [20].

Visual outcomes after airbag deployment are closely related to the severity of trauma: open-globe injuries and blunt trauma with posterior segment involvement represent the worst-case scenario. However, the most frequently reported injuries were eyelid wounds, corneal abrasions, and hyphema [24], which usually resolve without visual impairment.

Wearing glasses may play a role in shifting the type of injury: if they do not break during impact, they appear protective against blunt and chemical injuries. If they do break, they can lead to more severe penetrating injuries [25]. Some authors suggest that patients who have undergone eye surgery with corneal or scleral incisions may be more vulnerable to open-globe injury from blunt airbag trauma [14,26,27,28].

Although most eye trauma is unilateral, a broad spectrum of bilateral eye injuries has been described after airbag deployment [29,30,31,32,33,34,35,36,37,38,39,40,41,42,43,44,45]. The prevalence ranged from 12.5% [11] to 27% [45], and visual outcomes ranged from complete recovery [34] to bilateral absence of light perception [41].

## 2. Angle Recession and Post-Traumatic Glaucoma

Ocular trauma is a sight-threatening event. Vision loss can occur due to the eye trauma itself or due to its complications. Significant ocular hypertension is a major complication of traumatized eyes, which can lead to glaucoma over time. According to Girkin and colleagues, blunt trauma is more likely to cause glaucoma than penetrating trauma. The authors assessed the risk of glaucoma in two cohorts of patients from the United States Eye Injuries Registry. The first group experienced penetrating ocular trauma and had a 2.67% risk of developing glaucoma [46], while the second cohort suffered a non-penetrating eye injury and had a 3.39% risk of developing glaucoma [47].

Ocular hypertension can occur in the short or long term after trauma. Table 2 outlines the mechanisms of post-traumatic glaucoma with early and late onset.

### 2.1. Pathophysiology

Nowadays, angle recession is known as the most common cause of late-onset post-traumatic glaucoma [49]. Angle recession is defined as a pathological split between circular and longitudinal muscular fibers of the ciliary body. It was first observed by Edward Treacher Collins in 1890 in histological specimens of three eyes enucleated after concussion [50]. Wolff et al. in 1962 examined the late histopathological changes in these eyes. They noted significant atrophy of the detached circular ciliary muscle, diffuse fibrosis of the trabeculae, and obliteration of the Schlemm’s canal [51]. In many cases, a hyaline membrane, formerly described by Reese and D’Ombrain [52,53], covered the recessed angle, continuously with Descemet’s membrane. The authors identified the relationship between these anatomical findings and secondary glaucoma with insidious onset after ocular trauma [51].

The main features at gonioscopy are posterior displacement of the iris root, exposed ciliary band, and widening of the iridocorneal angle. In long-standing trauma, broad goniosynechiae may mask some of these findings. The development of goniosynechiae may be attributed to post-traumatic hyphema, iridocorneal contact, or more generically prolonged anterior chamber inflammation [54,55].

A Danish group precisely measured the widening of the iridocorneal angle in 276 eyes enucleated after trauma: the distance between the iris root and the scleral spur was 0.4 mm and between the iris and the Schwalbe line was 0.7 mm. Measurements in normal eyes were around 0.15 mm and 0.25 mm, respectively [56].

Pujari et al. hypothesized the probable mechanisms of angle recession. The anterior–posterior compression of the globe into the rigid structures of the orbit forcefully displaces the aqueous humor towards the iridocorneal angle: this acute stretching may exceed the elasticity of the tissues and cause damage to the angular structure [57], as previously reported by Duke-Elder [58]. Furthermore, the intermediate portion between the circular and longitudinal muscles of the ciliary body, containing oblique muscle fibers, is thinner and could be a zone of cleavage during trauma [57].

Eagling observed the correlation between the site of impact and the site of angle recession in 39 patients. In the case of limbal impact, angle recession was mainly found in the correspondent quadrant. Milder contrecoup damage in the opposite quadrant may occur. Only the opposite quadrant was involved when the impact was behind the limbus. In central corneal impact, extensive angle recession was observed [59]. Sectorial mydriasis may reveal angle recession in the corresponding quadrant due to localized damage of the nerve fibers which reach the pupillary sphincter muscle through the ciliary body [60].

The severity of angle recession is related to the circumferential extent and depth of the laceration. Mooney classified angle recession according to gonioscopic features (Table 3).

Although angle recession is relatively common after blunt ocular trauma, only 7–9% of patients develop post-traumatic glaucoma [62,63,64].

Blanton observed a bimodal pattern in the incidence of ocular hypertension after angle recession. The first peak occurs within 1 year of the ocular injury and seems unrelated to the extent of angle recession. In these cases, resistance to aqueous outflow is masked by ciliary body hyposecretion after trauma: ocular hypertension arises when the ciliary body regains its function and may be transitory. The second peak insidiously occurs several years after trauma and appears to be related to the extent of angle recession [62].

According to the literature, the greater the circumferential extent of angle recession, the greater the risk of developing post-traumatic glaucoma, but no exact correlations can be established [65,66]. Hyphema, trabecular pigmentation, lens dislocation, higher baseline IOP, and absence of cyclodialysis are other ocular findings significantly associated with glaucoma after closed-globe injury [67]. Furthermore, Girkin and colleagues found a significant association between increasing age, poor visual acuity at baseline, and the risk of glaucoma six months after injury [47].

### 2.2. Diagnosis

In clinical practice, the iridocorneal angle is assessed using either traditional or high-tech methods.

The traditional method is gonioscopy, where a goniolens is used to view the angle structures in detail. On the one hand, it is inexpensive, allows evaluation of pigmentation, and provides a dynamic assessment of the angle structures by indentation. On the other hand, it is a method that relies heavily on the operator’s experience and the patient’s cooperation and requires topical anesthesia and careful disinfection to avoid infection [68].

High-tech methods use anterior-segment optical coherence tomography (AS-OCT) or a Scheimpflug camera, which allow more objective measurement and precise follow-up, do not require an experienced operator, and do not expose patients to discomfort or risk of infection.

However, these instruments are much more expensive and cumbersome and do not allow qualitative assessment of angle structures (pigmentation or neovascularization) nor dynamic assessment of structures by indentation.

Therefore, gonioscopy and high-tech methods are considered complementary for the evaluation of the iridocorneal angle [68,69].

Angle recession can occur after blunt trauma either with or without other ocular findings, according to the severity of the trauma; hyphema is the most common association [60,62,64].

The prevalence of angle recession in the presence of hyphema is variable. Some authors report a very high prevalence ranging from 71% to 100% [62], while others report a lower prevalence of 23.5% [70].

It is interesting to note that angle recession can also occur without hyphema in cases where the trauma does not involve injury to the major blood vessels [71]. Patients may be unaware of their ocular injury and delay referral to an ophthalmologist [10] with a higher risk of late-onset complications, such as post-traumatic glaucoma.

### 2.3. Treatments

Post-traumatic glaucoma usually responds to medical therapy, but no scientific evidence supports a preference for one IOP-lowering agent over another. The only precaution is to avoid the use of prostaglandin analogues in the immediate post-trauma period, due to their possible pro-inflammatory effect [72].

When post-traumatic glaucoma is refractory to medical therapy, surgery is indicated. In filtration surgery, mitomycin-C is recommended to prevent fibrosis of the filtering bleb, which is common in eyes with previous trauma [68,73].

In addition to traditional trabeculectomy with mitomycin C, other surgical approaches have been tried for angle recession glaucoma. In recent research, Cheng et al. achieved satisfactory results with penetrating canaloplasty with good IOP control at 1 year [74]. Kaushik et al. achieved good results with primary implantation of the Ahmed valve with a 3-year follow-up [75]. Similarly, the efficacy of trabeculectomy in phakic eyes with angle recession was evaluated by Senthil and colleagues, with good results at 5 years even without mitomycin C [76]. Currently, no surgical glaucoma therapy has proven superior to others in refractory angle recession glaucoma, and further studies are needed to compare different surgical options in these cases.

## 3. Case Report

Angle recession due to airbag impact is not as uncommon as we might suppose.

Lesher and colleagues reported the first case in 1993 [77]; other cases have been described in adults and children [42,43,44]. Unfortunately, no extensive and detailed studies have been conducted on this topic. According to available data, the prevalence ranges from 11% to 15.4% [11,45], and bilateral presentation occurs up to 2.1% [24] (Table 4).

Eye trauma is considered one of the few causes of unilateral glaucoma, and bilateral presentation usually leads the clinician to rule out post-traumatic etiology. We reported the case of a 75-year-old Caucasian man with a bilateral presentation of angle recession. The patient’s medical history revealed a car accident 22 years earlier. He was driving wearing his seat belt; no injuries were reported, but his spectacles had been abruptly removed due to the airbag impact. Since then, he never had a complete ophthalmic evaluation.

**Figure 1:**
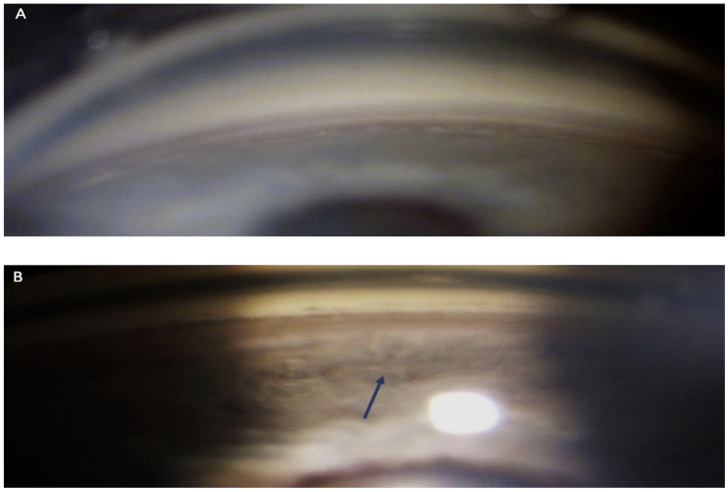
Right-eye non-indentation gonioscopy: superior quadrants (**A**), inferior quadrants (**B**). The inferior quadrants show a pathological deepening compared to the superior quadrants (arrow).

**Figure 2:**
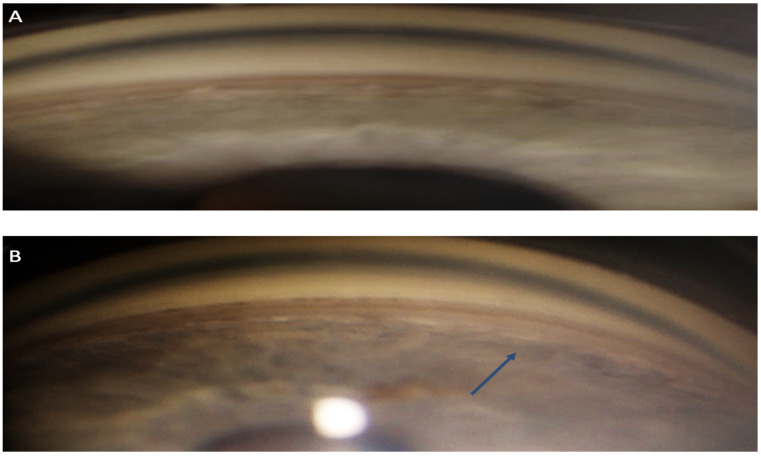
Left-eye non-indentation gonioscopy: superior quadrants (**A**), inferior quadrants (**B**). The inferior quadrants show a pathological deepening compared to the superior quadrants (arrow).

**Figure 3:**
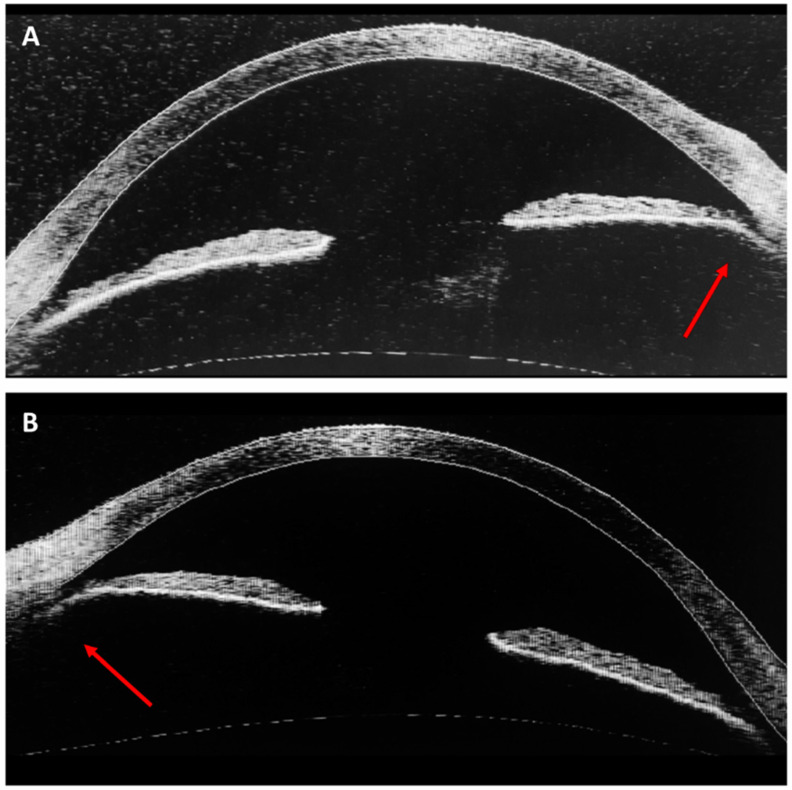
Anterior-segment OCT (Zeiss, Visante OCT—1000). Angle recession (arrows) in OD (**A**) and OS (**B**).

**Figure 4:**
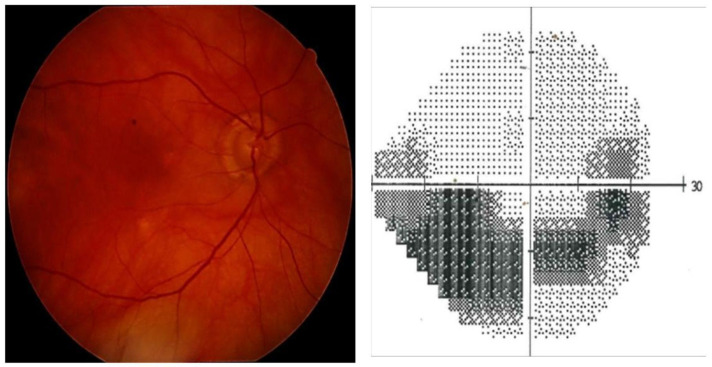
Right eye, fundoscopy and perimetry (Zeiss, HFA 24-2 SITA standard): advanced glaucoma (mean deviation—12.29 dB).

**Figure 5:**
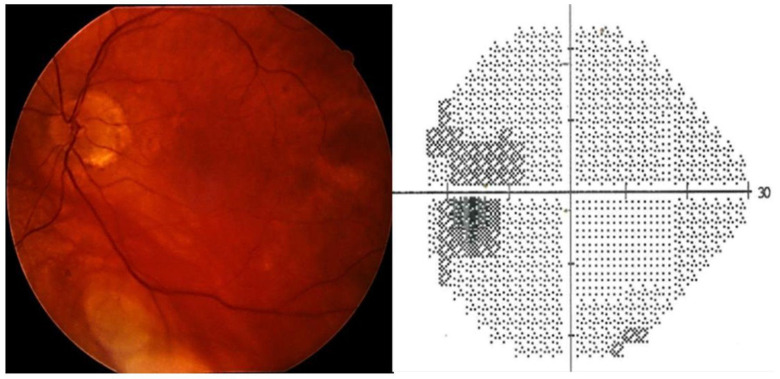
Left eye, fundoscopy and perimetry (Zeiss, HFA 24-2 SITA standard): peripapillary atrophy and corresponding blind-spot enlargement but no evident glaucoma damage.

The patient was referred to an ophthalmologist complaining of lateral visual defects. The best corrected visual acuity (BVCA) was 8/10 in the right eye (RE) and 7/10 in the left eye (LE). Intraocular pressure (IOP) with Goldman applanation tonometry was 35 mmHg in the RE and 25 mmHg in the LE. Slit lamp examination, gonioscopy, and AS-OCT showed mild iris atrophy and angle recession in both eyes (Figure 1, Figure 2 and Figure 3). Superior neuroretinal rim thinning was found in the RE (Figure 4) and no signs of glaucoma in the LE (Figure 5). Standard automated perimetry confirmed severe glaucoma defect in the RE, with inferior arcuate scotoma and superior nasal step (Figure 4).

IOP control and stability of the visual field defect were achieved in both eyes with medical therapy. This case drew attention to airbag-related ocular trauma as a possible cause of traumatic glaucoma with bilateral presentation.

## 4. Discussion

According to the literature, only 7–9% of patients with angle recession will develop glaucoma during their lifetime [42,43,44,77]. An extended follow-up is necessary to detect post-traumatic glaucoma because this long-term complication can occur even decades after trauma.

Airbag deployment is a known cause of eye trauma, and cases of unilateral and bilateral angle recession have been reported [42,43,44,77]. The prevalence of unilateral angle recession is up to 15.4% [45], and bilateral cases occur in up to 2.1% of people exposed to airbag-related eye injury [24]. Therefore, post-traumatic glaucoma after airbag deployment is an uncommon but possible occurrence. We want to emphasize that eye injuries, disability, and fatalities are more frequent in adults when the airbag is not part of the vehicle’s equipment [9,78], and we do not recommend disconnection, except in cases prescribed by safety regulations.

Along with angle recession, our patient also had a mild degree of iris atrophy and pigmentation of the trabecular meshwork. Some authors hypothesize a possible overlap between angle recession and pigment dispersion [79]. The retrocession of the iris diaphragm induced by the trauma would cause rubbing of the iris epithelium on the zonule and anterior capsule of the lens, similar to what occurs in pigment dispersion syndrome.

To our knowledge, we describe the first chronic post-traumatic glaucoma probably provoked by an airbag.

Other cases of airbag-induced angle recession have been reported; however, in the short term, IOP was controlled and long-term follow-up have not been reported [42,43,44,77].

Probably bilateral presentation of the angle recession may occur when a large object, such as an airbag, hits the whole face striking both eyes simultaneously.

Ocular and orbital rim prominence may be a risk factor and a protective factor, respectively, from airbag-related eye trauma [42,80].

Bilateral angle recession has also been described in other settings. A case of bilateral glaucoma, initially mistaken for congenital glaucoma, was reported in an abused child. The infant presented with multiple bilateral signs of ocular trauma and angle recession in the right eye. The authors were unable to identify angle recession in the left eye due to refractory corneal edema, but it could be supposed because the lens was subluxated and significant iridodialysis was present [81].

Similar considerations can be drawn from a population study conducted by Salmon in a South African village, with a high rate of interpersonal violence due to low socioeconomic status and alcohol abuse. Gonioscopy was performed in 983 people over 40 years: the prevalence of angle recession was high (14.8% *n* = 146). Among affected patients, bilateral involvement was widespread: 86 individuals had a bilateral presentation, and 60 individuals had a unilateral presentation [66]. This finding confirms that physical violence may be a possible cause of bilateral angle recession and a higher risk of blindness due to post-traumatic glaucoma. Bilateral occurrences of angle recession have been described in professional boxers [82,83] and could be expected in other contact sports.

The prevalence of unilateral and bilateral angle recession would probably increase if gonioscopy were routinely performed after blunt trauma by experienced ophthalmologists.

## 5. Conclusions

In our opinion, the traditional assertion that traumatic glaucoma is necessarily unilateral should be reconsidered. Although infrequent, cases of bilateral angle recession and/or traumatic glaucoma are possible and need long-term follow-up to avoid visual impairment. Airbag deployment, contact sports, or physical assault could be possible causes to be ruled out.

When the patient’s condition allows the examination, we suggest always performing bilateral gonioscopy after face and eye contusion. Even if only one eye is involved, comparison with the other could help reveal a subtle degree of angle recession. A careful medical history is recommended, focusing on possible causes of face trauma and other signs of physical abuse. This last consideration is utterly relevant in the case of minor patients.

## Figures and Tables

**Table 1:** Mechanical ocular trauma classification [3,4].

Closed globe	Contusion: blunt trauma without laceration of sclera and/or cornea.
	Lamellar laceration: partial thickness laceration of sclera and/or cornea.
	SOB *: foreign body embedded in the conjunctiva, cornea, or sclera without full thickness defect.
Open globe	Rupture: blunt trauma with full-thickness laceration of sclera and/or cornea.
	Penetration: full-thickness laceration of sclera and/or cornea due to a sharp object, without exit wound.
	IOFB **: foreign body retained after full-thickness laceration of sclera and/or cornea.
	Perforation: full-thickness laceration of sclera and/or cornea due to a sharp object, with an exit wound.
	Mixed injuries: a combination of the mechanisms above.

* SFB: superficial foreign body; ** IOFB: intraocular foreign body.

**Table 2:** Causes of early/late onset of post-traumatic glaucoma [48].

Post-traumatic glaucoma	
Early onset	Late onset
Trabeculitis/iritis	Angle recession
Trabecular disruption	Peripheral anterior synechiae
Hyphema	Ghost cell glaucoma
Massive choroidal hemorrhage	Lens-related *
Chemical injury	Delayed closure of cyclodialysis cleft
	Epithelial downgrowth
	Retained intraocular foreign body
	Rhegmatogenous retinal detachment (Schwartze syndrome)

* Lens-related glaucoma includes lens dislocation, lens swelling, phacolytic glaucoma, and lens particle glaucoma.

**Table 3:** Grading of angle recession [61].

Grade	Gonioscopy Description
Grade I Shallow angle tear	No definite ciliary cleft: ciliary band is darker and wider than in the other eye
Grade II Moderate angle tear	Definite ciliary cleft: the angle is deeper than that of the other eye due to a tear of the ciliary body
Grade III Deep angle tear	Deep fissure into the ciliary body: the apex of the fissure cannot be identified gonioscopically

**Table 4:** Angle recession due to airbag deployment.

Author	Angle Recession *n* (%)	Bilateral Angle Recession *n* (%)	Comment
Lesher, M.P. (1993) [77]	-	-	Case report; unilateral angle recession, associated to corneal edema and hyphema
Driver, P.J. (1994) [42]	-	-	Letter to editor; bilateral angle recession and hyphema
Michaeli Cohen, A. (1995) [43]	-	-	Letter to editor; bilateral angle recession, corneal contusion and hyphema
Ghafouri, A. (1997) [45]	6 (15.4%)	0 (0%)	Case series and review of the literature; 32 patients with airbag-related ocular injury
Stein, J.D. (1999) [24]	11 (11.3%)	2 (2.1%)	Review of the literature; 97 patients with airbag-related ocular injury
Pearlman, J.A. (2001) [11]	11 (11%)	2 (2%)	Review of the literature; 101 patients with airbag-related ocular injury
Alquraini, T.A. (2010) [44]	-	-	Case report, 14-year-old patient; bilateral angle recession, hyphema and corneal abrasion, left vitreous hemorrhage and macular edema

## Data Availability

All data analyzed during this study are included in this published article.

## References

[1] Négrel A.D., Thylefors B. (1998). The global impact of eye injuries. Ophthalmic Epidemiol..

[2] Ng S.M.S., Low R., Hoskin A.K., Rousselot A., Gunasekeran D.V., Natarajan S., Sundar G., Chee C.K.L., Mishra C., Sen P. (2022). The application of clinical registries in ophthalmic trauma—The International Globe and Adnexal Trauma Epidemiology Study (IGATES). Graefes Arch. Clin. Exp. Ophthalmol..

[3] Kuhn F., Morris R., Witherspoon C.D., Heimann K., Jeffers J.B., Treister G. (1996). A Standardized Classification of Ocular Trauma. Ophthalmology.

[4] Pieramici D.J., Sternberg P., Aaberg T.M., Bridges W.Z., Capone A., Cardillo J.A., De Juan E., Kuhn F., Meredith T.A., Mieler W.F. (1997). A System for classifying mechanical injuries of the eye (Globe). Am. J. Ophthalmol..

[5] Bhalla K., Gleason K. (2020). Effects of vehicle safety design on road traffic deaths, injuries, and public health burden in the Latin American region: A modelling study. Lancet Glob. Health.

[6] International Transport Forum (2016). Road Safety Annual Report 2016.

[7] International Transport Forum (2018). Road Safety Annual Report 2018.

[8] Motley W.W., Kaufman A.H., West C.E. (2003). Pediatric airbag-associated ocular trauma and endothelial cell loss. J. Am. Assoc. Pediatr. Ophthalmol. Strabismus.

[9] Braver E.R., Ferguson S.A., Greene M.A., Lund A.K. (1997). Reductions in deaths in frontal crashes among right front passengers in vehicles equipped with passenger air bags. JAMA.

[10] Wallis L.A. (2002). Injuries associated with airbag deployment. Emerg. Med. J..

[11] Pearlman J.A., Eong K.G.A., Kuhn F., Pieramici D.J. (2001). Airbags and eye injuries. Surv. Ophthalmol..

[12] Lee W.B., O’Halloran H.S., Pearson P.A., Sen H.A., Reddy S.H.K. (2001). Airbags and bilateral eye injury: Five case reports and a review of the literature. J. Emerg. Med..

[13] Malhotra K., Rose J., Homer N. (2022). Devastating injury from blunt airbag trauma. J. Am. Coll. Emerg. Physicians Open.

[14] Duma S.M. (2002). The effect of frontal air bags on eye injury patterns in automobile crashes. Arch. Ophthalmol..

[15] Shirzadi H., Zohoor H., Naserkhaki S. (2018). Biomechanical simulation of eye-airbag impacts during vehicle accidents. Proc. Inst. Mech. Eng..

[16] Fukagawa K., Tsubota K., Kimura C., Hata S., Mashita T., Sugimoto T., Oguchi Y. (1993). Corneal Endothelial Cell Loss Induced by Air Bags. Ophthalmology.

[17] Duma S.M., Kress T.A., Porta D.J., Simmons R.J., Alexander C.L., Woods C.D. (1997). Airbag-induced eye injuries: Experiments with in situ cadaver eyes. Biomed. Sci. Instrum..

[18] Kobayashi A., Izaki R., Fujita H., Harada K., Ozaki H., Kadonosono K., Uchio E. (2023). Finite element analysis of changes in tensile strain and deformation by airbag impact in eyes of various axial lengths. Int. Ophthalmol..

[19] Huang J., Uchio E., Goto S. (2015). Simulation of airbag impact on eyes with different axial lengths after transsclerally fixated posterior chamber intraocular lens by using finite element analysis. Clin. Ophthalmol..

[20] Azukisawa M., Sato T., Ito Y., Kamezawa H., Nomura E., Nishide T., Kadonosono K., Uchio E., Goto S. (2005). Simulation of airbag impact on eyes after trabeculectomy by finite element analysis method. Nippon Ganka Gakkai Zasshi.

[21] Uchio E., Watanabe Y., Kadonosono K., Matsuoka Y., Goto S. (2003). Simulation of airbag impact on eyes after photorefractive keratectomy by finite element analysis method. Graefes. Arch. Clin. Exp. Ophthalmol..

[22] Uchio E., Kadonosono K., Matsuoka Y., Goto S. (2004). Simulation of air-bag impact on an eye with transsclerally fixated posterior chamber intraocular lens using finite element analysis. J. Cataract. Refract. Surg..

[23] Uchio E., Ohno S., Kudoh K., Kadonosono K., Andoh K., Kisielewicz L.T. (2001). Simulation of air-bag impact on post-radial keratotomy eye using finite element analysis. J. Cataract. Refract. Surg..

[24] Stein J.D., Jaeger E.A., Jeffers J.B. (1999). Air bags and ocular injuries. Trans. Am. Ophthalmol. Soc..

[25] Lehto K.S., Sulander P.O., Tervo T.M.T. (2003). Do motor vehicle airbags increase risk of ocular injuries in adults?. Ophthalmology.

[26] Goldberg M.A., Valluri S., Pepose J.S. (1995). Air Bag-related Corneal Rupture After Radial Keratotomy. Am. J. Ophthalmol..

[27] Maharshak I., Bourla D., Grinbaum A., Weinberger D., Axer-Siegel R. (2005). Airbag-induced bilateral corneal graft dehiscence. Cornea.

[28] Uchio E., Kadonosono K. (2001). Are airbags a risk for patients after radial keratotomy?. Br. J. Ophthalmol..

[29] Zacovic J.W., McGuirk T.D., Knoop K.J. (1997). Bilateral hyphemas as a result of air bag deployment. Am. J. Emerg. Med..

[30] Salam T., Stavrakas P., Wickham L., Bainbridge J. (2010). Airbag injury and bilateral globe rupture. Am. J. Emerg. Med..

[31] Ogun O., Ikyaa S., Ogun G. (2014). Rethinking airbag safety: Airbag injury causing bilateral blindness. Middle East Afr. J. Ophthalmol..

[32] Subash M., Manzouri B., Wilkins M. (2010). Airbag-induced chemical eye injury. Eur. J. Emerg. Med..

[33] Welch J.F. (2013). Bilateral chemical eye injury resulting from airbag deployment. J. R. Nav. Med. Serv..

[34] Fante R.J., Trobe J.D. (2014). Bilateral corneal abrasions from airbag deployment. N. Engl. J. Med..

[35] Onwuzuruigbo C.J., Fulda G.J., Larned D., Hailstone D. (1996). Traumatic blindness after airbag deployment: Bilateral lenticular dislocation. J. Trauma Inj. Infect. Crit. Care.

[36] Odouard C., Kuo C., Tariq Y.M., Ha J.H., Swamy B. (2016). Central visual loss following a motor vehicle accident: Traumatic airbag maculopathy. Med. J. Aust..

[37] Ball D.C., Bouchard C.S. (2001). Ocular morbidity associated with airbag deployment: A report of seven cases and a Review of the Literature. Cornea.

[38] Savastano A., Donati M.C., Rizzo S. (2016). Retinal tear related to air bag deployment. JAMA Ophthalmol..

[39] Lueder G. (2000). Air bag-associated ocular trauma in children. Ophthalmology.

[40] Ingraham H.J. (1991). Air-Bag Keratitis. N. Engl. J. Med..

[41] Criado A.L., López P.B., Alonso A.G. (2010). Permanent visual loss secondary to airbag deployment. Acta Ophthalmol..

[42] Driver P.J., Cashwell L.F., Yeatts R.P. (1994). Airbag-associated bilateral hyphemas and angle recession. Am. J. Ophthalmol..

[43] Michaeli-Cohen A., Neufeld M., Lazar M., Geyer O., Haddad R., Kashtan H. (1996). Bilateral corneal contusion and angle recession caused by an airbag. Br. J. Ophthalmol..

[44] Alquraini T.A., Aggour M.A., Zamzam A.M. (2011). Airbag induced facial and bilateral ocular injuries in a 14-year-old child. Saudi J. Ophthalmol..

[45] Ghafouri A., Burgess S.K., Hrdlicka Z.K., Zagelbaum B.M. (1997). Air bag-related ocular trauma. Am. J. Emerg. Med..

[46] Girkin C.A., McGwin G., Morris R., Kuhn F. (2005). Glaucoma following penetrating ocular trauma: A cohort study of the United States Eye Injury Registry. Am. J. Ophthalmol..

[47] Girkin C.A., McGwin G., Long C., Morris R., Kuhn F. (2005). Glaucoma after ocular contusion: A cohort study of the United States eye injury registry. J. Glaucoma.

[48] Yanoff M., Duker J.S. (2014). Ophthalmology.

[49] Tumbocon J.A.J., Latina M.A. (2002). Angle recession glaucoma. Int. Ophthalmol. Clin..

[50] Collins E.T. (1890). On the Pathological Examination of Three Eyes Lost from Concussion.

[51] Wolff S.M., Zimmerman L.E. (1962). Chronic secondary glaucoma. Associated with retrodisplacement of iris root and deepening of the anterior chamber angle secondary to contusion. Am. J. Ophthalmol..

[52] Reese A.B. (1944). Deep-chamber glaucoma due to the formation of a cuticular product in the filtration Angle. Trans. Am. Ophthalmol. Soc..

[53] D’Ombrain A. (1949). Traumatic or “concussion” chronic glaucoma. Br. J. Ophthalmol..

[54] Walton W., Von Hagen S., Grigorian R., Zarbin M. (2002). Management of traumatic hyphema. Surv. Ophthalmol..

[55] Loo Y., Tun T.A., Vithana E.N., Loo Y., Tun T.A., Vithana E.N., Perera S.A., Husain R., Wong T.T., Aung T. (2021). Association of peripheral anterior synechiae with anterior segment parameters in eyes with primary angle closure glaucoma. Sci. Rep..

[56] Jensen O.A. (2009). Contusive angle recession. A histopathological study of a danish material. Acta Ophthalmol..

[57] Pujari A., Selvan H., Behera A.K., Gagrani M., Kapoor S., Dada T. (2020). The probable mechanism of traumatic angle recession and cyclodialysis. J. Glaucoma.

[58] Duke-Elder S. (1972). Mechanical Injuries.

[59] Eagling E.M. (1974). Ocular damage after blunt trauma to the eye. Its relationship to the nature of the injury. Br. J. Ophthalmol..

[60] Tönjum A.M. (1966). Gonioscopy in traumatic hyphema. Acta Ophthalmol..

[61] Mooney D. (1972). Anterior chamber angle tears after non-perforating injury. Br. J. Ophthalmol..

[62] Blanton F.M. (1964). Anterior chamber angle recession and secondary glaucoma: A study of the aftereffects of traumatic hyphemas. Arch. Ophthalmol..

[63] Kaufman J.H., Tolpin D.W. (1974). Glaucoma after traumatic angle recession a ten-year prospective study. Am. J. Ophthalmol..

[64] Mooney D. (1973). Angle recession and secondary glaucoma. Br. J. Ophthalmol..

[65] Alper M.G. (1963). Contusion angle deformity and glaucoma: Gonioscopic observations and clinical course. Arch. Ophthalmol..

[66] Salmon J.F., Mermoud A., Ivey A., Swanevelder S.A., Hoffman M. (1994). The detection of post-traumatic angle recession by gonioscopy in a population-based glaucoma survey. Ophthalmology.

[67] Sihota R. (2008). Early predictors of traumatic glaucoma after closed globe injury: Trabecular pigmentation, widened angle recess, and higher baseline intraocular pressure. Arch. Ophthalmol..

[68] European Glaucoma Society (2021). Terminology and Guidelines for Glaucoma, 5th Edition. Br. J. Ophthalmol..

[69] Jindal A., Ctori I., Virgili G., Lucenteforte E., Lawrenson J.G. (2020). Non-contact tests for identifying people at risk of primary angle closure glaucoma. Cochrane Database Syst. Rev..

[70] Mowatt L., Chambers C. (2010). Ocular Morbidity of traumatic hyphema in a Jamaican Hospital. Eur. J. Ophthalmol..

[71] Capão Filipe J.A., Barros H., Castro-Correia J. (1997). Sports-related Ocular Injuries. Ophthalmology.

[72] Shah C., Sen P., Tabani S., Prasad K., Peeush P., Jain E. (2023). Clinical features of early-onset pediatric traumatic glaucoma and predictive factors for the need of early glaucoma surgery. Indian J. Ophthalmol..

[73] Bai H.Q., Yao L., Wang D.B., Jin R., Wang Y.X. (2009). Causes and Treatments of Traumatic Secondary Glaucoma. Eur. J. Ophthalmol..

[74] Cheng H., Ye W., Zhang S., Xie Y., Gu J., Le R., Deng Y., Hu C., Zhao Z., Ke Z. (2023). Clinical outcomes of penetrating canaloplasty in patients with traumatic angle recession glaucoma: A prospective interventional case series. Br. J. Ophthalmol..

[75] Kaushik J., Parihar J.K.S., Singh A., Shetty R., Singhal A., Chaitanya Y.V.K., Jain V.K., Mathur V. (2022). Evaluation of primary Ahmed Glaucoma valve implantation in post-traumatic angle recession glaucoma in Indian eyes. Int. Ophthalmol..

[76] Senthil S., Dangeti D., Battula M., Rao H.L., Garudadri C. (2022). Trabeculectomy with mitomycin-C in post-traumatic angle recession glaucoma in phakic eyes with no prior intraocular intervention. Semin. Ophthalmol..

[77] Lesher M.P. (1993). Corneal Edema, Hyphema, and angle recession after air bag inflation. Arch. Ophthalmol..

[78] Anderson S.K., Desai U.R., Raman S.V. (2002). Incidence of ocular injuries in motor vehicle crash victims with concomitant air bag deployment. Ophthalmology.

[79] Qing G., Wang N., Wang H. (2012). Pigment dispersion secondary to anterior chamber angle recession. Graefes Arch. Clin. Exp. Ophthalmol..

[80] Bhavsar A.R., Chen T.C., Goldstein D.A. (1997). Corneoscleral laceration associated with passenger-side airbag inflation. Br. J. Ophthalmol..

[81] Tseng S.S., Keys M.P. (1976). Battered child syndrome simulating congenital glaucoma. Arch. Ophthalmol..

[82] Palmer E., Lieberman T.W., Burns S. (1976). Contusion angle deformity in prizefighters. Arch. Ophthalmol..

[83] Giovinazzo V.J., Yannuzzi L.A., Sorenson J.A., Delrowe D.J., Cambell E.A. (1987). The ocular complications of boxing. Ophthalmology.

